# Step-by-step optimisation of robotic-assisted radical prostatectomy using augmented reality

**DOI:** 10.1590/S1677-5538.IBJU.2022.99.10

**Published:** 2022-01-12

**Authors:** Jonathan Noël, Marcio Covas Moschovas, Ela Patel, Travis Rogers, Jeffrey Marquinez, Bernardo Rocco, Alexandre Mottrie, Vipul Patel

**Affiliations:** 1 AdventHealth Global Robotics Institute United States of America AdventHealth Global Robotics Institute, Urology, Celebration, United States of America; 2 Università degli Studi di Milano Milan Italy Università degli Studi di Milano, Urology, Milan, Italy; 3 OLV Hospital ORSI Academy Melle Belgium OLV Hospital ORSI Academy, Urology, Melle, Belgium

## Abstract

**Introduction:**

Surgical training will be complemented by digitalisation, as the COVID 19 pandemic continues (1). Proximie is an augmented reality (AR) platform that can display up to 4 native camera views, with live or semi live telementoring. It can optimise ergonomics of the surgeon at the console (2), and robotic instrument orientation. We describe the utilisation of Proximie as a step-by-step guide in a robotic assisted radical prostatectomy (RARP).

**Surgical Technique:**

Author V. P. performed a transperitoneal multiport da Vinci Xi RARP with the Proximie platform: a laptop computer, multiple HD webcams, microphones and speakers. Using an HDMI cable to the Intuitive Surgical tower, output display from the console and an additional laparoscopic tower is shown. Each webcam was mounted to the side armrests of the console, directed at the surgeon's hands. An independent ‘drop in’ laparoscope via an additional 5mm left upper quadrant port was utilised. Observers can visualise the AR platform's recordings on a laptop and/or smartphone. A PTZ (pan-tilt-zoom) camera can capture the operating room, bedside assistant, ports and patient position. Our video demonstrates three of four camera views for posture, forearm, wrist, hand, and finger orientation, relative to the translated robotic steps. A pincer grasp of the endowrist manipulator during anastomosis allows optimal robotic wrist rotation. The second laparoscopic camera view demonstrated intracorporeal angles of robotic arm and bedside assistant's instrument position for critical steps such as nerve sparing and anastomosis (3). The console time was 100 minutes, no intraoperative complications, or delay in image transmission occurred with utilising the platform.

**Considerations:**

An AR platform can create deeper learning for RARP in real time or recorded sessions. Two-way verbal and visual communication with ability to annotate on screen, allows long distance mentoring. The platform's utility can be accessed in anywhere, to project surgeons beyond their immediate environment. This allows for democratisation of access to high volume institutions and their evolution of techniques (4), to assist patients globally. Potential developments are artificial intelligence (AI) networks analysing repository of such recorded data, to identify intraoperative hand motion and robotic instrument tracking. AR is a pertinent building block to enhance robotic training, skill dissemination, precision medicine (5) and surgery overall.
